# Prolactin, TNF alpha and nitric oxide expression in nitroso-N-methylurea-induced-mammary tumours

**DOI:** 10.1186/1477-3163-6-18

**Published:** 2007-11-28

**Authors:** Irene Vegh, Rafael Enríquez de Salamanca

**Affiliations:** 1 Centro de Investigacion, Hospital Universitario 12 de Octubre. Madrid, Av. Cordoba s/n, CP, 28041, Spain

## Abstract

**Background:**

The N-Nitrosomethylurea breast cancer model induced in rats is used for the study of carcinogenesis in mammary cancer, prostate, pancreas, etc. This model is very similar to human neoplastic disease.

**Methods:**

The present experimental study was designed to assess whether metoclopramide administration has any effect on development of MNU-induced tumours, and evaluate the treatment of goserelin acetate on PRL, TNF alpha and NO expression. NMU was administered to female Wistar rats on 2 occasions (5 mg/100 g body w/rat). PRL and TNF alpha were performed by immune-assay. Nitric Oxide by semi automated-assay and ploidy analyses by flow cytometry.

**Results:**

The administration of metoclopramide made the induction time shorter and increased the incidence and average of tumours per rat. Tumours development was inhibited by a goserelin chronic administration. The ploidy of adenocarcinoma was polyploid-aneuploid type (average S = 60%). It was higher basal PRL plasma levels in rats with NMU induced tumours than in basal controls without tumour (p < 0.001). The goserelin "in bolus" administration showed maximal inhibition of plasma PRL at 90 min. Plasmatic TNF alpha expression was inhibited at 60 min and also remained inhibited in tissue homogenate post chronic treatment (P < 0.0125). Plasmatic NO expression is higher in rats with induced tumours than healthy controls (P < 0.001). In tissue homogenate NO values were inhibited at 90 min (P < 0.01), as well during chronically goserelin treatment (P < 0.005).

**Conclusion:**

The increase of blood PRL levels in NMU-induced rats may be an indicator of a poor prognosis of mammary cancer evolution. The metoclopramide administration accelerates tumour growth. However goserelin administration achieves regression in tumour development associated to inhibition PRL, TNF alpha and NO expression.

## Background

N-nitroso-N-methylurea (NMU) is an effective chemical carcinogen for the induction of mammary carcinoma in rats and a very good model for human mammary carcinomas [1-6]. Prolactin (PRL) is a mitogenic hormone similar to growth factors and rat mammary tumour express PRL messenger RNA and acts as a local growth factor that stimulates proliferation of mammary tumours [7]. The presence of specific receptors for PRL has been demonstrated and they belong to the cytokine family of receptors [8-10]. PRL may act as a positive growth factor in mammary tumour development and increase the proliferation of breast cancer cells in rats [7].

Metoclopramide (C_14_H_22_ClN_3_O_2_), their IUPAC name: 4-amino-5-chloro-N-(2-diethylamino)ethyl-2-methoxybenzamide is a potent dopamine receptor antagonist used for its antiemetic effect and by inhibiting the action of PRL inhibiting hormone and has sometimes been used to stimulate lactation. It thus may induce hyperprolactinemia in rats.

The agonistic analogue of gonadotrophin-releasing hormone (LH-RH) is known to suppress ovarian function and plasma prolactin levels and its antitumour activity has been evaluated extensively [11].

In chemical carcinogenesis, internal cytokines, such as interleukin (IL) 1α and tumour necrosis alpha (TNFα) contribute to tumour progression. However it has been suggested that TNF on tumour vasculature has a direct anti-tumour mechanism [12-14]. Indeed estradiol has inhibitory effects on IL 1α and TNFα mRNA and their proteins expression [15].

Inducible nitric oxide synthase (iNOS) and nitric oxide (NO) are implicated in tumour pathology and, it is well known that TNFα induces iNOS and NO release. While low concentrations of NO alter transcription factor and act as an antioxidant factor, high concentrations of NO can damage DNA and proteins. Moreover NO-induced alteration in cellular function can lead to cell death or stimulation of growth, and stimulate tumour growth by promoting angiogenic and invasive capacities [16-19].

The aim of the study was to assess the effect of metoclopramide on tumour development of NMU-induced tumours in Wistar rats, and at the same time to study acute and chronic administration of (LH-RH analogue) goserelin on PRL, TNFα and NO expression in this experimental model.

## Methods

### Animals

Wistar rats were used for all the experiments and were obtained at 45 days of age. Animals were housed 2/cage in a room illuminated for 12 h each day at a temperature of 25°C. The animals had free access to Purina laboratory chow and drinking water. All the experiments in this study were performed in compliance with the Royal Decree 223/1988 of March (BOE 8 18) and the Ministerial Order of 13 October 1989 (BOE 8) regarding protection of the experimental animals, as well as with the European Council Directive of 24 November 1986 (86/609/EEC).

### Chemicals

Crystalline NMU (Sigma, No. N1517) was dissolved in 0.85% NaCl solution and acidified (acetic acid) at 5.0 pH. The concentration was then adjusted to 5 mg/100 g body wt/rat. Heparin-treated blood (0.2 ml) was obtained by cardiac puncture from rats lightly anesthetized with ether.

### Tumour detection

Animals were palpated for detection of mammary tumours initialling after injection of NMU and the animals were examined by palpation for tumour masses thrice weekly post administration of carcinogen. Tumour sizes were measured with a calliper and their growth and histological parameters were tested.

### Histology

The diagnoses of mammary gland cancer were made by histological examination of paraffin sections stained with haematoxylin/eosin in all tumours. Autopsy was performed when the neoplasms were large. A nodule of mammary cancer is designated an active tumour, the number of active tumours is expressed per rat with mammary cancer.

### Preparation of tumour fraction and blood samples

The tumour samples were prepared in the following manner: after sacrifice of the animals by decapitation the tumours were removed immediately. All further steps in the preparation were performed at 0–4°C. After being washed in ice-cold PBS buffer (pH 7.4) the tumours were homogenized in this buffer (10 ml/5 g of wet tumour). The homogenate was isolated by centrifugation for 30 min at 9000 × g and the supernatant were stored in aliquots at -80°C until used in each of the corresponding assays.

### Measurement of prolactin, TNF α and NO concentration in plasma and mammary tumour homogenate

Tumour homogenate and plasma were analyzed for prolactin concentration using a commercially available ELISA kit (PRL, kit ELISA, Amersham Pharmacia Biotech, Barcelona, Spain). The basal level of rat plasma was in accord to that indicated and obtained using standard rat plasma provided by the ELISA kit and indicating that rat stress was avoided, as confirmed by the variability of the plasma blood levels in controls.

For TNF α concentration was using a commercially available kit: (Factor-Test™, mTNF-α ELISA Test Kit, Genzyme, Spain).

An assay for Nitric Oxide concentration was performed according to Navarro-Gonzáles et al [20].

The blood was centrifuged at 1500 g for 15 min; plasma was harvested and stored at -80°C until the hormonal concentration and biomarkers concentration were measured.

Total protein concentration was determined by the Lowry assay (Bio-Rad, Madrid, Spain) for tumour homogenates. In homogenate TNF α, NO concentrations were expressed relative to total protein.

Histological analysis was performed on primary NMU tumours on haematoxylin-eosin stained sections. Flow cytometry (FACScan, Becton Dickinson) was performed of rat NMU induced tumours. Flow cytometry was performed on nuclei prepared from 40 μm thick sections from formalin fixed paraffin tissue by the modified technique of Hedley et al. and McLemore et al [21,22].

### Experimental Design

At 55 days of age, the female rats were divided into the following groups:

1- Healthy Controls (n = 10) without NMU-induction.

2- NMU controls (n = 20) received weekly an injection of NMU [a set of 2 injections during 2 weeks NMU (5 mg/100 g weight/rat)] and was observed after carcinogen administration for tumour development.

3- NMU plus metoclopramide (M) (n = 20) in drinking water (0.125 mg/L), "at libitum", was observed weekly for tumour development. The elimination half-life for (M) is 5–6 hours.

4- NMU plus goserelin (n = 40) and the metoclopramide administration was suspended in this group when tumour was detected and they were subdivided into 2 groups for (a) acute and (b)chronic study, respectively:

a) Received goserelin administration "in bolus" (0.25 mg/ml/rat, i.v.) and killed 2 h after injection.

b) Received goserelin (subcutaneous/daily) during 60 days (0.25 mg/ml/day).

### Statistical Analysis

Differences in tumour incidence were demonstrated using x^2 ^analysis and Yates correction. Inter-group comparisons were performed by Student t test. Values of p < 0.05 were considered to be significantly different.

## Results

Post administration of NMU in control group, mammary cancer was detected in 9 of 20 (45%) rats on days 90–120. Metoclopramide treatment had effect on NMU-induced tumours, where the incidence was 85%. Latency to the appearance of first palpable tumour tended to be shorter in all rats that received metoclopramide. Indeed the average of tumours was higher than in controls, Table 1.

**Table 1:** Metoclopramide (M) administration on NMU tumours.

Wistar rats	Induction time (days)	Incidence (%)	Average of tumours
Control NMU	90 – 120	45%	0.5 t/rat
NMU + M	55	85%	1.4 t/rat

NMU = N-nitroso-N-methylurea. M = metoclopramide. t = tumour. The tumour induction time, tumour incidence and average of tumours were increased in rats with metoclopramide administration.

**Figure 1:**
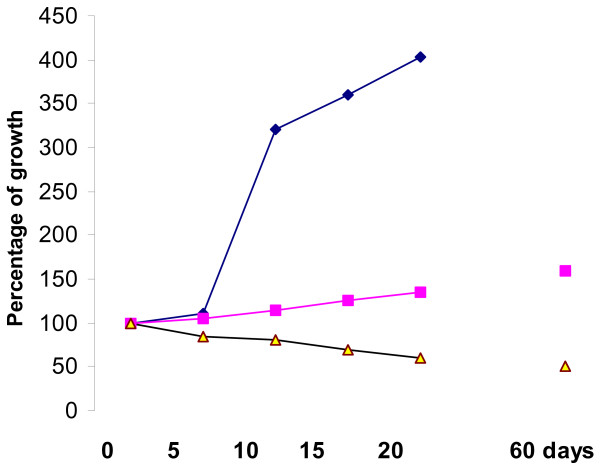
Tumours evolution post-NMU administration (0–60 days) post-first measurement (= 100% as initial size)-: -■- NMU control rats without treatment -◆- NMU tumours treated with metoclopramide. -▲- NMU tumours treated with goserelin.

In NMU-induced tumours plus (M) the size of tumours observed an increase in growth at 400% on 20^th ^day, compared with the administered goserelin, where the tumours stop in their development or diminish in size (last one observed at 60^th ^day, Fig 1.

The histology revealed that all the tumours were adenocarcinoma.

**Figure 2:**
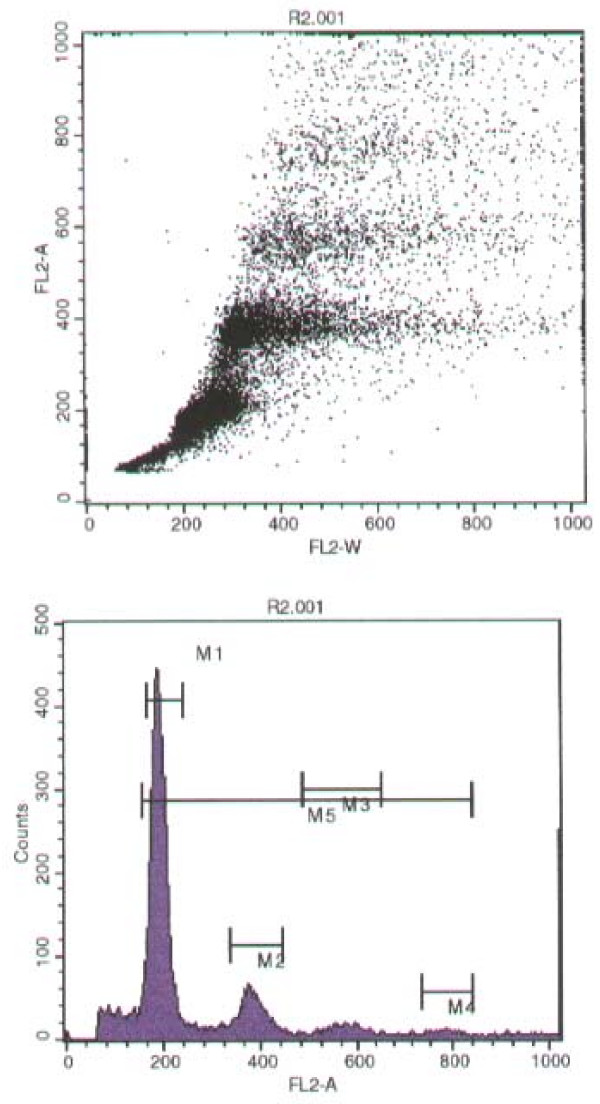
Flow cytometry analyses of NMU induced mammary tumours. The dot plot images (top) and histogram (bottom) of the NMU-induced adenocarcinoma sample. They observed polyploidy distribution in all samples.

Analyses by flow cytometry of nuclear DNA patterns revealed 100% polyploidy in NMU induced tumours. According to this study all tumour samples were polyploidy-aneuploid and none of them was diploid type, percentage of S = 60%, Mitosis (ratio) = 1.99. DNA Index = 1.50 – 2.00, and with a CV (G0/G1) = 9%. Fig 2.

### PRL measurements

**Figure 3:**
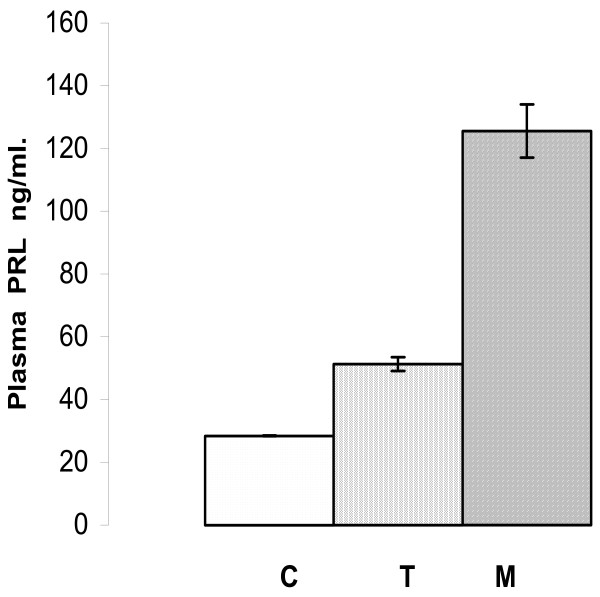
Immunoreactive plasma PRL means levels. In healthy controls (C) without NMU-induced tumours were lower than rats with NMU-induced tumours (T), and lower than with metoclopramide treatment (M). (Mean ± SEM).

In **plasma **the mean value of PRL levels was lower in healthy controls [C] (28.5 ± 0.1 ng/ml, n = 20) than in rats with NMU tumours [T] (51.3 ± 2.2 ng/ml, n = 20), P < 0.01. During metoclopramide (M) administration the PRL basal mean levels were 125.6 ± 8.5 ng/ml, n = 20 in healthy controls, Fig 3.

The PRL plasma mean levels of NMU tumour rats [T], were higher than in tumour homogenates [TH] (corresponding to the same group of rats and extraction time) 5.8 ± 1.2 ng/ml (n = 20); these results were statistically significant (P < 0.0005).

The PRL plasma levels decreased in NMU rats when they were treated with goserelin. During acute administration the time course was, basal: 51.3 ± 2.2 ng/ml (n = 20); 30 min 26.2 ± 6.5 ng/ml (n = 20), P < 0.001; 60 min 17.5 ± 13.5 ng/ml (n = 20), P < 0.0125; and at 90 min: 12.2 ± 4.2 ng/ml (n = 20), P < 0.0005.

**Figure 4:**
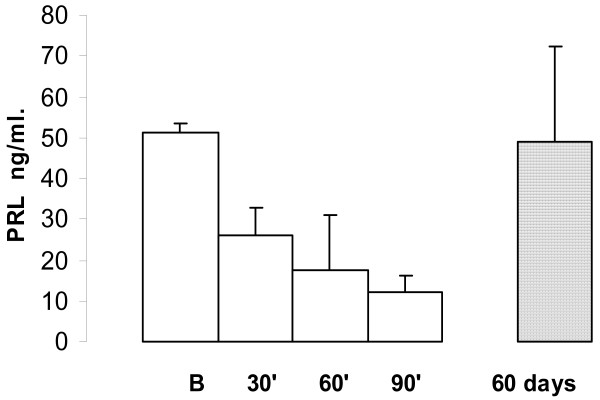
The plasma PRL time course during goserelin administration. In the NMU induced mammary tumours the values observed maximal inhibition at 90 minutes (P < 0.005). PRL value in chronically treated rats with goserelin was similar to basal (B) value; each point represents the mean ± SEM.

During chronic administration of goserelin the mean PRL levels were similar to those in healthy control rats: 49.0 ± 23.4 ng/ml (n = 20) P = 0.40, Fig 4.

In **tumour homogenate **[TH] there was no statistically significant variations of PRL mean levels during acute administration of goserelin neither post chronically treated: Basal 5.8 ± 1.2 ng/ml (n = 20); 30 min 6.9 ± 0.1 ng/ml (n = 20), P = 0.20; 60 min 5.6 ± 0.3 ng/ml (n = 20); at 90 min 6.8 ± 0.2 ng/ml (n = 20), P = 0.35. At 60 days treatment the mean value was 5.9 ± 0.1 ng/ml (P = 0.35).

### TNF alpha measurements

**Figure 5:**
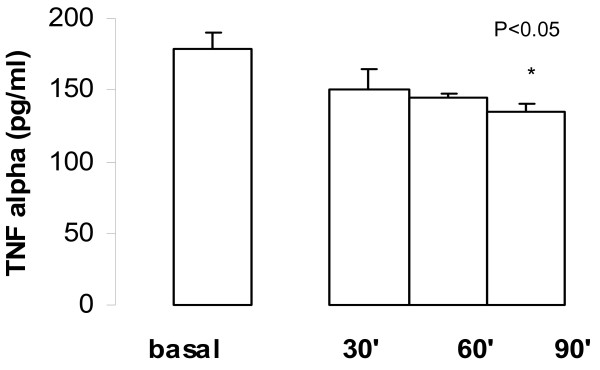
Time course of TNF α expression in plasma during acute administration of goserelin. The values observed maximal inhibition at 90 min from basal values; each point represents the mean ± SEM.

TNFα expression during time course of acute administration of goserelin observed inhibition in **plasma**. The mean values were: Basal 178.7 ± 11.6 pg/ml (n = 20); at 30 min 150.0 ± 15.0 pg/ml (n = 20), P = 0.10; at 60 min 145.2 ± 2.5 pg/ml (n = 20), P < 0.05; and at 90 min. 135.0 ± 5.0 (n = 20), P < 0.05; Fig 5.

The inhibition of TNF α expression was observed during post-chronic treatment with goserelin: 132.1 ± 52.2 pg/ml, n = 20 (P < 0.0125).

In **tumour homogenates **mean basal values of TNFα were: 255.0 ± 30.0 pg/mg of protein (n = 20), at 30 min 210.0 ± 12.5 pg/mg of protein (n = 18), P = 0.20; at 60 min 200.0 ± 15.0 pg/mg of protein (n = 18), P < 0.05; and at 90 min 210.0 ± 15.0 pg/mg of protein (n = 18), P = 0.10.

**Figure 6:**
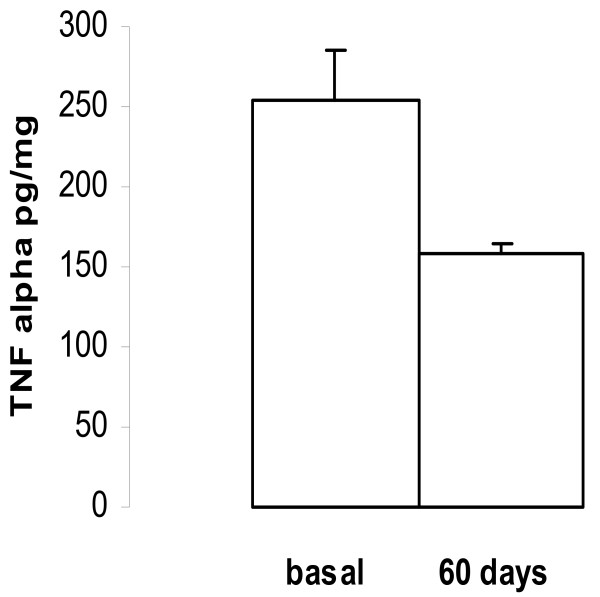
Effect of goserelin on TNF α concentration in tumour homogenate. At 60 days of LH-RH treatment, the mean value of TNF α was lower than basal value (P < 0.0125) (mean ± SEM).

At 60 days of goserelin treatment, the TNFα expression observed a statistically significant inhibition when compared with the basal values, 255.0 ± 30.5 vs. 159.0 ± 5.2 pg/mg of protein (n = 18), P < 0.0125, Fig 6.

### Nitric Oxide measurement

**Figure 7:**
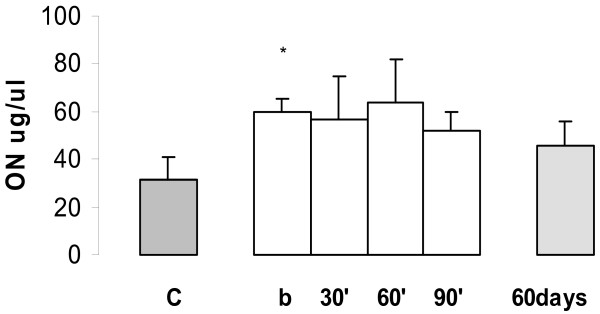
Expression of NO in plasma in the NMU-induced mammary tumours. Healthy control without tumours (C) versus basal (b) NMU induced rats *P < 0.001 (Values expressed as mean ± SEM). During the time course for NO expression acute and chronic administration of goserelin, between them the differences were not statistically significant.

In female Wistar rats without NMU induced tumour, the basal mean NO concentration in plasma was 31.5 ± 9.5 μg/μl, n = 20. The mean NO concentrations were higher in plasma of female Wistar rats with NMU induced tumours than basal healthy controls (59.6 ± 5.5 μg/μl n = 20, P < 0.001). In **plasma **inhibition of NO expression during time course of acute administration of goserelin has been observed but these differences were not statistically significant. Basal mean values were: 59.6 ± 5.5 μg/μl (n = 20), 30 min 56.7 ± 17.8 μg/μl (n = 20), P = 0.10; at 60 min 64.0 ± 17.9 μg/μl (n = 20) P = 0.15 and at 90 min 52.2 ± 7.5 μg/μl (n = 20) P = 0.25. Lack of inhibition of NO expression by goserelin was observed during chronic treatment 46.0 ± 19.9 μg/μl, n = 20 (P = 0.25) when compared with NMU induced basal values, Fig 7.

The NO concentration was inhibited by goserelin acute treatment in **tumour homogenate**. The mean basal concentration was: 205.9 ± 42.3 μg/μg of protein (n = 20), at 30 min 134.0 ± 17.9 μg/μg of protein (n = 20) P = 0.05, at 60 min: 118.0 ± 22.9 μg/μg of protein (n = 20), P < 0.05, and at 90 min. 82.3 ± 9.9 μg/μg of protein (n = 20), P < 0.01.

**Figure 8:**
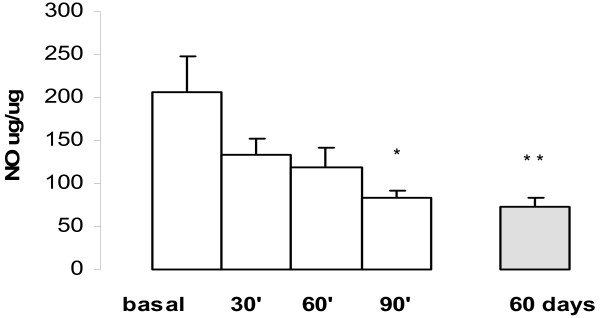
Concentration of NO in NMU-induced tumour homogenate during administration of goserelin (time course). The maximal inhibition of NO was at 90 min (*P < 0.01) and remained at 60 days (** P < 0.005), each point represents the mean ± SEM.

In chronically administered goserelin (at 60 day) the NO values lowered to 72.4 ± 9.9 μg/μg of protein and when compared with basal NO values statistically significant differences was observed between them (P < 0.005), Fig 8.

## Discussion

The MNU breast cancer model induced in rats is used for the study of carcinogenesis in mammary cancer, prostate, pancreas, etc. This model is very similar to human neoplastic disease [23]. The developmental stage of mammary gland at the time of exposure of the gland to NMU has an important effect of the gland to carcinogenesis [1].

A great variability of various mouse and rat strains for sensitivity on mammary tumours with chemical initiating agents was studied and considered that their results support the need to use more then one rat strain for the initiation of mammary carcinogenesis, due to "very low"-sensitivity for the NMU tumour development in Wistar-Han rats [24]. Nevertheless, in Wistar rats the incidence of tumours per rat and percentage of rats with NMU tumours was lower than observed in Sprague-Dawley rats as observed in a previous study. In our experiments we administered intra-peritoneal carcinogen at twice and in both studies the rats received metoclopramide daily, as inductor of PRL release and, as has been demonstrated PRL acts as positive growth factor in mammary tumour development suggesting that the hormonal mileu around the time of carcinogen exposure affects not only the incidence and phenotype of mammary transformants but also the molecular events associated with mammary carcinogenesis [3,7,25]. Experimental studies proposed that PRL could act as a local growth factor and may stimulate the proliferation of mammary NMU-induced tumours. [7,10,26].

Our study demonstrated that metoclopramide (as dopaminergic antagonist) has a very high capacity to induce hyperprolactinemia in rat, indeed the tumour growth accelerated during the observation period and this effect could be influenced or stimulated by PRL release. These results are in agreement with different previous experimental and clinical studies on PRL-induced proliferation. [9,27-33]. Thus sulpiride a selective dopamine D2 antagonist has been documented in a clinical study the administration of LH-RH inhibits sulpiride-induced hyperprolactinemia [34].

The histological study of our tumours revealed adenocarcinoma type, similar to observed in human breast cancer. Flow cytometric S-phase fraction contributes to diagnosis of tumours. At present to our knowledge, only flow cytometric analyses were performed in NMU-induced solid tumours in rat prostate [35]. Monakov et al [36] observed chromosomal damages and polyploidy and hiperpolyploid in somatic cells after the NMU administration and considered that they can serve as a prognostic factor of carcinogenesis. Our study is the first communication about the flow cytometry analysis of the NMU-induced tumours in female rats and it showed polyploidy (aneuploid) in all the tumour samples. These results contributed to discriminate from possible benign-induced tumours since benign tumours would be diploid type.

Initially an acute administration of LH-RH agonist induces a release of LH and FSH. However, continuous and chronic administration produces an inhibition of the hypophyseal-gonadal-axis though the process of "down regulation" of pituitary receptors for LH-RH. Furthermore the expression on LH-RH receptor gene was established in tumours [37]. In our study the tumour growth was inhibited by chronically administration of goserelin and an important tumour regression was observed at 60 days of treatment. During chronic administration of goserelin the mean PRL levels were similar to that in healthy control rats, in this way previous metoclopramide-induced hyperprolactinemia and by tumour presence, PRL expression was inhibited. During our study we observed an increase of plasmatic TNF α in rats with NMU-induced and estradiol dependent tumours when compared to controls without NMU-induced tumours. Moreover, it is well known that a variety of chemical carcinogens can adversely affect the immune system and influence tumour evolution and exert toxic effect on lymphoid organs, thus possibly affecting the production of cytokines at the initiation of carcinogenesis. This increment was inhibited by LH-RH analogues treatment and according to Tagami et al. may act by inhibition of estrogens production, because estrogens are also responsible of COX-2 induction, the same way as c-fos, IL-1 and TNF alpha [38].

It is well known that NO is a free radical that mediates a variety of cell functions. The plasmatic NO expression of our study showed a statistically significant increase of NO concentration in rats with NMU-induced tumours when compared to controls, which was observed in basal conditions. These results suggest that NO can stimulate tumour growth. Additionally, TNFα induces iNOS and NO production and could mediate tumour promoter-induced transformation.

In this study we have examined PRL, TNF alpha and NO levels in plasma and in tumour homogenates during goserelin administration and, similar behaviour was observed. PRL levels in homogenate presented no statistical significant differences. It could be due to the initial low values in homogenate or because the sensitivity of the assay was insufficient.

It is well known that NO levels in cancer cells contribute to tumour angiogenesis and during acute administration of goserelin it did not completely inhibit this increase. At present, the practical importance of these values or relevance with respect to predicting the disease course is not clear.

This study demonstrates firstly the role "in vivo" of metoclopramide on tumour growth and secondly for goserelin in NMU-induced mammary tumours in which inhibitory effects were observed on growth as well as with significant tumour regression, besides it also exerted inhibitory effects on plasmatic and tissular PRL, TNFα and ON expression. In summary, these findings could reveal a possible new inhibitory pathway in the tumour development regulation by LH-RH analogues.

## Authors' contributions

IV designed and carried out the experiments and wrote the manuscript and REdeS participated in study analysis and drafted the manuscript. All authors read and approved the final manuscript.
